# A glutathione-dependent control of the indole butyric acid pathway supports Arabidopsis root system adaptation to phosphate deprivation

**DOI:** 10.1093/jxb/eraa195

**Published:** 2020-04-20

**Authors:** José A Trujillo-Hernandez, Laetitia Bariat, Tara A Enders, Lucia C Strader, Jean-Philippe Reichheld, Christophe Belin

**Affiliations:** 1 Université Perpignan Via Domitia, Laboratoire Génome et Développement des Plantes, UMR, Perpignan, France; 2 CNRS, Laboratoire Génome et Développement des Plantes, UMR, Perpignan, France; 3 NSF Science and Technology Center for Engineering Mechanobiology, Department of Biology, Washington University in St. Louis, St. Louis, MO, USA; 4 University Paris sud, France

**Keywords:** Arabidopsis, auxins, glutathione, indole butyric acid, lateral roots, phosphate, root hair, root system

## Abstract

Root system architecture results from a highly plastic developmental process to adapt to environmental conditions. In particular, the development of lateral roots and root hair growth are constantly optimized to the rhizosphere properties, including biotic and abiotic constraints. The development of the root system is tightly controlled by auxin, the driving morphogenic hormone in plants. Glutathione, a major thiol redox regulator, is also critical for root development but its interplay with auxin is scarcely understood. Previous work showed that glutathione deficiency does not alter root responses to indole acetic acid (IAA), the main active auxin in plants. Because indole butyric acid (IBA), another endogenous auxinic compound, is an important source of IAA for the control of root development, we investigated the crosstalk between glutathione and IBA during root development. We show that glutathione deficiency alters lateral roots and root hair responses to exogenous IBA but not IAA. Detailed genetic analyses suggest that glutathione regulates IBA homeostasis or conversion to IAA in the root cap. Finally, we show that both glutathione and IBA are required to trigger the root hair response to phosphate deprivation, suggesting an important role for this glutathione-dependent regulation of the auxin pathway in plant developmental adaptation to its environment.

## Teaser

Arabidopsis root responses to indole butyric acid (IBA), but not IAA, depend on glutathione levels, and endogenous IBA and glutathione are both required for root system adaptation to phosphate starvation.

## Introduction

Root developmental plasticity, combining root growth and root branching, is essential for plants to adapt and optimize their growth in changing environmental conditions, such as nutrient and water availability, rhizosphere microbiome, or soil structure heterogeneity. Root growth relies on the activity of the root apical meristem that regulates histogenesis via the control of cell proliferation. Root branching is a complex organogenesis process that allows the development of new lateral roots (LRs) from regularly spaced pericycle founder cells in *Arabidopsis thaliana*. These founder cells are originally specified by an oscillatory mechanism occurring in the root tip (Moreno-Risueno *et al.*, 2010; Xuan *et al.*, 2016). In addition to the root system architecture, the development of epidermal root hairs (RHs) is particularly sensitive to changes in environmental conditions, and they contribute to the root system adaptation (Grierson *et al.*, 2014).

Among nutrients, phosphorus has a key role in all living organisms and is one of the main limiting factors for plant growth and crop productivity (Leinweber *et al.*, 2018). Its homeostasis is highly dependent on phosphate uptake by the root cap (Kanno *et al.*, 2016). Phosphate concentration strongly impacts root system development in many plant species including Arabidopsis (López-Bucio *et al*., 2002, 2003; Hodge, 2004). In particular, general low phosphate availability is well known to increase RH length (Bates and Lynch, 1996). Biotic factors also modulate root development. Among them, some rhizospheric PGPR (plant growth-promoting rhizobacteria) such as *Mesorhizobium loti* are able to stimulate RH elongation, hence favouring soil exploration and nutrient uptake by the plant (Vacheron *et al.*, 2013; Poitout *et al.*, 2017).

Auxins, and particularly the most abundant endogenous auxin, indole acetic acid (IAA), play a pivotal and integrative role in all steps of root system development, under both optimal and stress conditions (Saini *et al.*, 2013; Korver *et al.*, 2018). During LR development, auxin is responsible for the activation of founder cells, the development and organization of the LR primordia (LRPs), and the emergence of the newly formed LRs through the external layers of the primary root. Even earlier, the oscillatory positioning of pericycle founder cells depends on auxin maxima generated via auxin release by dying root cap cells (Moreno-Risueno *et al.*, 2010; Du and Scheres, 2018). Auxin also modulates RH elongation, thus mediating responses to stimuli such as abscisic acid or phosphate deprivation (Nacry *et al.*, 2005; Wang *et al.*, 2017). Recently, a set of works on Arabidopsis and rice evidenced that RH response to phosphate deprivation requires an increase in auxin *de novo* synthesis in the root cap, and its apico-basal transport to the epidermal cells via the auxin influx facilitator AUX1 (Bhosale *et al.*, 2018; Giri *et al.*, 2018; Parry, 2018).

Indole-3-butyric acid (IBA) is a structural derivative of IAA, differing by only two additional carbons in the side chain (Korasick *et al.*, 2013). Although convincing evidence supports a role for IAA in IBA biosynthesis, the mechanisms responsible and enzymes involved are still unknown (Ludwig-Müller *et al.*, 1995; Frick and Strader, 2018). It is now broadly accepted that IBA solely acts as an IAA precursor through its β-oxidative decarboxylation in peroxisomes (reviewed in Frick and Strader, 2018). This enzymatic process involves several enzymes, some shared with other β -oxidation pathways such as PED1, and others apparently specific to the IBA to IAA conversion, such as IBR1, IBR3, IBR10, and ECH2 (Strader and Bartel, 2011; Frick and Strader, 2018). IBA homeostasis is also regulated via its transport and conjugation, but only a few regulators have been identified. The type G ABC transporters ABCG36 and ABCG37 can efflux IBA from the cells (Strader and Bartel, 2009; Ruzicka *et al.*, 2010), the NPF family member TRANSPORTER OF IBA1 transports IBA across the vacuolar membrane (Michniewicz *et al.*, 2019), while the generic PXA1/COMATOSE transporter is likely to import IBA into the peroxisome. Like other auxins, IBA conjugates with sugars and amino acids. UGT84B1, UGT74D1, UGT74E2, and UGT75D1 can conjugate IBA to glucose (Jackson *et al.*, 2001; Tognetti *et al.*, 2010; Jin *et al.*, 2013; Zhang *et al.*, 2016), whereas GH3.15 is able to conjugate IBA to amino acids (Sherp *et al.*, 2018). IBA-derived IAA plays important roles during root development, including root apical meristem maintenance and adventitious rooting. IBA conversion to IAA is also critical for RH elongation (Strader *et al.*, 2010). Finally, recent works have reported the critical role of IBA to IAA conversion in the root cap as a source of auxin for the oscillatory positioning of LR founder cells (De Rybel *et al.*, 2012; Xuan *et al.*, 2015). IBA to IAA conversion taking place in the LRP itself also probably participates in further LR development (Strader and Bartel, 2011; Michniewicz *et al.*, 2019). Despite all these reported functions in root development, the importance of IBA-derived IAA relative to other IAA sources is still poorly understood and scarcely documented, particularly in the case of changing environmental constraints.

Changes in environmental conditions result in reactive oxygen species (ROS) imbalance, thus affecting the general redox cellular homeostasis (Noctor *et al.*, 2018). For example, phosphate deficiency alters H_2_O_2_ and O_2_^**·**–^ production in roots (Tyburski *et al.*, 2009). ROS modulate many aspects of plant development, including root system development (Singh *et al.*, 2016; Tsukagoshi, 2016). Controlled ROS production by NADPH oxidases is particularly involved in RH elongation and LR development (Carol and Dolan, 2006; Mangano *et al*., 2016, 2018; Orman-Ligeza *et al.*, 2016). Auxin and ROS pathways interact to control root development, since auxin can trigger ROS production and in turn ROS can affect IAA levels and transport (Orman-Ligeza *et al.*, 2016; Tognetti, 2017; Zwiewka *et al.*, 2019).

Glutathione is a small and stable thiol-containing tripeptide (Glu–Cys–Gly) essential for plant survival and present in large concentrations in cells, up to several millimolar (Noctor *et al.*, 2012). It has many roles, including the detoxification of heavy metals and xenobiotics, sulfur homeostasis, ROS homeostasis, and redox signalling. To ensure these functions, glutathione can act as a precursor (e.g. of phytochelatins), be conjugated to other molecules via the glutathione *S*-transferase enzyme family, or serve as an electron donor for antioxidant systems (Noctor *et al.*, 2012). Reduced glutathione (GSH) is therefore converted to oxidized glutathione (GSSG) which is reduced back by glutathione reductases (Marty *et al*., 2009, 2019). In standard growth conditions, GSH is in large excess relative to GSSG. Glutathione biosynthesis is a two-step reaction, with *GSH1* encoding the rate-limiting γ-glutamylcysteine synthetase and *GSH2* encoding a glutathione synthetase (Meyer *et al.*, 2012; Noctor *et al.*, 2012). Knock-out mutations in either of these two genes are lethal. However, genetic screens identified several knock-down alleles of *GSH1*, allowing plants with reduced glutathione levels to be obtained (Howden *et al.*, 1995; Vernoux *et al.*, 2000; Ball *et al.*, 2004; Parisy *et al.*, 2007; Jobe *et al.*, 2012; Shanmugam *et al.*, 2012). The importance of glutathione in root development is illustrated by the absence of root meristem maintenance in the *rml1* mutant, together with the impairment of primary root growth and LR development in less severe *gsh1* alleles (Vernoux *et al.*, 2000; Bashandy *et al.*, 2010; Koprivova *et al.*, 2010; Marquez-Garcia *et al.*, 2014).

We previously reported that *cad2* or *pad2* mutants can respond almost normally to exogenous IAA for root development (Bashandy *et al.*, 2010), although auxin transport seems to be affected in glutathione-deficient plants (Bashandy *et al.*, 2010; Koprivova *et al.*, 2010). The role of glutathione in controlling root development is therefore still misunderstood. Given the importance of IBA-derived IAA in regulating root development, we chose to address the role of glutathione in root responses to IBA. We show that glutathione deficiency impairs LR and RH responses to IBA but not IAA. We then try to identify the glutathione-dependent mechanisms required for IBA responses, but every IBA-related pathway examined does not reveal sensitivity to glutathione levels. We finally suggest a physiological role for this glutathione-dependent IBA response, showing that IBA and glutathione pathways are critical for RH responses to phosphate deficiency.

## Materials and methods

### Plant material

All plants used throughout this study are in the *Arabidopsis thaliana* Col-0 ecotype. The following mutants were all previously published and available in our teams: *cad2-1* (Howden *et al.*, 1995), *pad2-1* (Parisy *et al.*, 2007), *zir1* (Shanmugam *et al.*, 2012), *gr1* (Mhamdi *et al.*, 2010), *ntra ntrb* (Bashandy *et al.*, 2010), *cat2* (Bueso *et al.*, 2007), *ibr1-2* (Zolman *et al.*, 2008), *ibr3-1* (Zolman *et al.*, 2007), *ibr10-1* (Zolman *et al.*, 2008), *ibr1 ibr3 ibr10* (Zolman *et al.*, 2008), *ech2* (Strader *et al.*, 2011), *aux1-21* (Bennett *et al.*, 1996), and *pin2/eir1-1* (Roman *et al.*, 1995). *pdr8/pen3-4* (Stein *et al.*, 2006), *pdr9-2* (Ito and Gray, 2006), *ugt74e2* (Tognetti *et al.*, 2010), and *ugt74d1* (Jin *et al.*, 2013) are previously described Salk T-DNA insertion lines (Salk_000578, Salk_050885, Salk_091130, and Salk_011286, respectively) ordered from the Nottingham Arabidopsis Stock Centre (NASC; http://arabidopsis.info/). The double mutants *pdr8 pdr9* and *ugt74d1 ugt74e2* were obtained by crossing the respective single mutants, and by selecting the double homozygotes among F_2_ plants.

### Plant cultures

Seeds were surface sterilized by constant agitation with 0.05% SDS in 70% (v/v) ethanol for 20 min, then washed three times with 95% (v/v) ethanol and dried on sterile paper. Seeds were placed on plates containing 50 ml of half-strength Murashige and Skoog (1/2 MS) medium with 0.5 g l^–1^ MES and 0.8% (w/v) plant agar (Duchefa Biochemie) without sucrose, unless indicated. For the experiments regarding phosphate deprivation, we used three-tenths strength (3/10) MS supplemented with 500 µM NaCl (phosphate deprivation) or 500 µM NaH_2_PO_4_ (control conditions) as previously published (Arnaud *et al.*, 2014). For *M. loti* experiments, seeds were grown on standard 1/2 MS medium for 5 d, then transferred to new plates containing 0.1 OD of inoculum (Poitout *et al.*, 2017) for an extra 4 d before phenotyping. All plates were incubated vertically at 20 °C with 160 µE m^–2^ s^–1^ light intensity and a 16 h light/8 h dark regime.

### Phenotypic analyses of the root system

LR density was quantified on 10-day-old seedlings by counting the number of visible LRs emerged and dividing by the length of the primary root section between the first and the last visible LRs. LRP density was calculated in the same way on 6-day-old seedlings using a light microscope (Axioscop2, Zeiss) for counting. For gravistimulation experiments, LRP initiation was forced by rotating the vertical plates by 90°. LRP stages were observed 48 h later, using a light microscope (Axioscop2, Zeiss). For quantification of RH lengths, plates were photographed using a camera (DFC425C, Leica) sited on a stereomicroscope (MZ12, Leica). The primary root elongation rate was quantified between day 8 and day 10. Lengths were quantified from pictures using the public domain image analysis program ImageJ 1.52i (https://imagej.nih.gov/ij/) and its NeuronJ plugin (Meijering *et al.*, 2004).

### Glutathione measurements

Total glutathione content of 8-day-old seedlings was determined using the recycling enzymatic assay (Rahman *et al.*, 2006). The method consists of the reduction of GSSG by glutathione reductase and NADPH to GSH. GSH levels are determined by its oxidation by 5,5′-dithio-bis(2-nitrobenzoic acid) (DTNB), that produces a yellow compound 5′-thio-2-nitrobenzoic acid (TNB), measurable at 412 nm (Rahman et al., 2006). Briefly, 100 mg of fresh plant material ground in liquid nitrogen was extracted in 0.5 ml of 0.1 M Na phosphate buffer, pH 7.6, and 5 mM EDTA. After microcentrifugation (10 min, 9000 *g*), total glutathione in 0.1 ml of the supernatant was measured by spectrophotometry in a 1 ml mixture containing 6 mM DTNB (Sigma-Aldrich), 3 mM NADPH, and 2 U of glutathione reductase from *Saccharomyces cerevisiae* (Sigma-Aldrich). Glutathione-dependent reduction of DTNB was followed at 412 nm. Total glutathione levels were calculated using the equation of the linear regression obtained from a standard GSH curve. GSSG was determined in the same extracts after derivatization of reduced GSH. Derivatization of 100 ml of plant extract was performed in 0.5 ml of 0.5 M K phosphate buffer, pH 7.6, in the presence of 4 ml of 2-vinylpyridine (Sigma-Aldrich) during 1 h at room temperature. After extraction of the GSH-conjugated 2-vinylpyridine with 1 vol. of diethylether, GSSG was measured by spectrophotometry as described for total glutathione.

### Confocal analyses

Auxin response analyses using *DR5:n3EGFP/DR5v2:ntdTomato* (Liao *et al.*, 2015) and the study of the redox state of glutathione with the roGFP2 line (Meyer *et al.*, 2007) were performed by using a confocal laser scanning microscope LSM 700 (Zeiss). Images were acquired in 16 bits using a ×10 EC Plan Neofluar objective (Zeiss). Settings were based on the respective methods previously published (Meyer *et al.*, 2007; Liao *et al.*, 2015) with minor modifications. Excitation of roGFP2 was performed at 488 nm and 405 nm, and a bandpass (BP 490–555 nm) emission filter was used to collect the roGFP2 signal. For background subtraction, the signal was recovered using a BP 420–480 nm emission filter during excitation at 405 nm.

For *DR5:n3EGFP/DR5v2:ntdTomato* analysis, seeds were grown on standard 1/2 MS medium supplemented or not with 0.5 mM buthionine sulfoximine (BSO; pre-treatment). Seven-day-old seedlings were transferred for 24 h to plates containing the appropriate treatments (10 µM IBA or 50 nM IAA), still combined or not with 0.5 mM BSO, according to the pre-treatment. Regarding the analyses with roGFP2, seeds were grown for 8 d in 1/2 MS medium with or withoutt 10 µM IBA.

Picture analyses and quantifications were performed as previously described for both probes (Meyer *et al.*, 2007; Liao *et al.*, 2015), using the public domain image analysis program ImageJ 1.52i (https://imagej.nih.gov/ij/).

### Histochemical localization of β -glucuronidase (GUS) activity

Plants were fixed in 80% (v/v) acetone at 20 °C for 20 min, then washed with buffer solution, containing 25 mM Na_2_HPO_4_, 25 mM NaH_2_PO_4_, 2% Triton X-100 (v/v), 1 mM K_3_Fe(CN)_6_, and 1 mM K_4_Fe(CN)_6_. Thereafter, staining was performed at 37 °C in the buffer solution containing 2 mM X-Gluc (5-bromo-4-chloro-3-indolyl-β-d-glucuronidase) as substrate (Jefferson *et al.*, 1987), after 1 min vacuum infiltration. The reaction was stopped by changing the seedlings to 70% (v/v) ethanol. Pictures were collected using a light microscope (Axioscop2, Zeiss).

### Gene expression quantification

Total RNA was extracted using TriZol reagent (GE Healthcare, UK), and the RNA was purified with the RNeasy Plant Mini Kit (Promega, USA), according to the manufacturer’s protocols. cDNA was subsequently synthesized using the GoScript™ Reverse Transcription System (Promega, USA). Quantitative real-time PCR was done using Takyon™ No Rox SYBR® MasterMix blue dTTP (Eurogentec, Belgium) and the LightCycler 480 (Roche, Switzerland). Primers used are presented in Supplementary Table S1 at *JXB* online. All reported results are presented normalized with the *ACTIN2* control gene but behave similarly if normalized with *ACTIN7* or *GAPDH* control genes.

### Auxin accumulation assays

Root tips (5 mm) from 8-day-old seedlings were excised and incubated in 40 µl of uptake buffer (20 mM MES, 10 mM sucrose, and 0.5 mM CaSO_4_, pH 5.6) for 15 min at room temperature. Root tips were then incubated for 1 h in uptake buffer containing 25 nM [^3^H]IAA (20 Ci mmol^–1^; American Radiolabeled Chemicals) or [^3^H]IBA (25 Ci mmol^–1^; American Radiolabeled Chemicals) prior to washing with three changes of uptake buffer. Root tips were then placed in 3 ml of CytoScint-ES liquid scintillation cocktail (MP Biomedicals) and analysed by scintillation counting.

### Data replicability and statistical analyses

All the presented experiments illustrate results obtained in at least three independent biological repetitions. For *in vitro* phenotyping experiments, each biological repetition consisted of two technical replicate plates per condition, each containing ~12 seeds for the mutant and 12 for the appropriate control, sown side by side. Statistical analyses were performed as indicated in the figure legends.

### Accession numbers

Sequences from this article can be found in the Arabidopsis Genome Initiative database with the following accession numbers: *GSH1* (*At4g23100*), *GR1* (*At3g24170*), *NTRA* (*At2g17420*), *NTRB* (*At4g35460*), *CAT2* (*At4g35090*), *AUX1* (*At2g38120*), *PIN2* (*At5g57090*), *PDR8/PEN3* (*At1g59870*), *PDR9* (*At3g53480*), *UGT74D1* (*At2g31750*), *UGT74E2* (*At1g05680*), *IBR1* (*At4g05530*), *IBR3* (*At3g06810*), *IBR10* (*At4g14430*), *ECH2* (*At1g76150*), *AIM1* (*At4g29010*), *PED1* (*At2g33150*), *PHT1;4* (*At2g38940*), *RNS1* (*At2g02990*), *SPX1* (*At5g20150*), and *ACT2* (*At3g18780*).

## Results

### Glutathione deficiency specifically alters LR and RH responses to IBA but not IAA

Since IBA plays important roles during root development, we investigated root responses to both IAA and IBA in several *gsh1* weak alleles and in plants treated with BSO, a specific chemical inhibitor of GSH1.

**Fig. 1.**
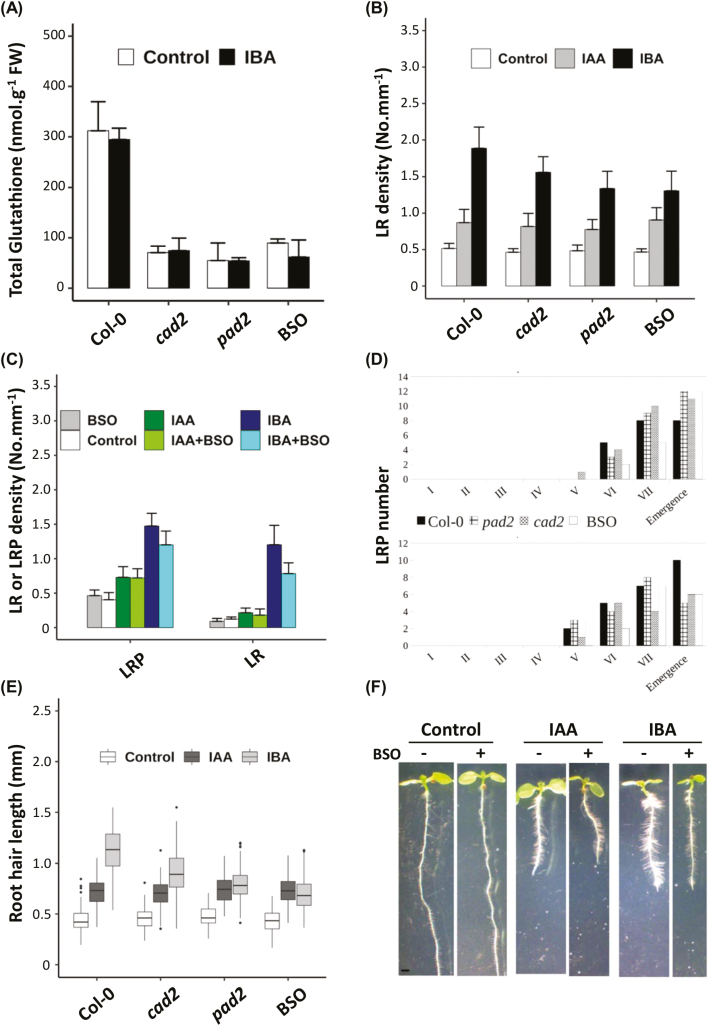
Glutathione specifically regulates root responses to IBA. (A) Total glutathione content in 8-day-old wild-type (Col-0), *cad2*, or *pad2* seedlings grown on standard 1/2 MS medium. Wild-type plants grown in the presence of 0.5 mM BSO were also assayed. Data represent the means of three biological repetitions. (B) Emerged lateral root density of 10-day-old wild-type (Col-0), *cad2*, or *pad2* plants grown on standard 1/2 MS medium and wild-type plants grown in the presence of 0.5 mM BSO (*n*>15). (C) Emerged LR and LRP density of 10-day-old wild-type plants grown on standard 1/2 MS medium (control), or in the presence of different combinations of 0.5 mM BSO, 10 µM IBA, and/or 50 nM IAA, as mentioned (*n*>15). (D) Developmental stages of LRPs of 6-day-old wild-type (Col-0), *cad2*, or *pad2* seedlings 48 h after gravistimulation on standard 1/2 MS medium supplemented (bottom panel) or not (top panel) with 10 µM IBA; wild-type plants grown in the presence of 0.5 mM BSO were also assayed. Data indicate the percentage of LRPs in each developmental stage (*n*>16) and are representative of three independent experiments. (E) Pictures of representative 8-day-old wild-type plants grown on standard 1/2 MS medium, or in the presence of different combinations of 0.5 mM BSO, 10 µM IBA, and/or 50 nM IAA, as mentioned. (F) Quantification of root hair length of 8-day-old wild-type (Col-0), *cad2*, or *pad2* mutant plants grown on standard 1/2 MS medium (Control), or in the presence of 50 nM IAA or 10 µM IBA. Wild-type plants in the presence of 0.5 mM BSO were also assayed (*n*>100). Histograms represent the mean, and error bars represent the SD. Asterisks indicate a significant difference, based on a two-tailed Student *t*-test (**P≤0.001).

We first wanted to know the precise glutathione levels in the different genotypes in our growth conditions, thus we quantified endogenous glutathione in whole 8-day-old seedlings (Fig. 1A). We find the same amount of total glutathione (i.e. ~25% of the wild-type content) in *cad2* and *pad2* mutants, which was expected. We show that 1 mM exogenous BSO also reduces endogenous glutathione levels of wild-type plants to approximately the same levels. In addition, we report that exogenous IBA treatment does not impact endogenous glutathione levels.

Supplementary Fig. S1A and B shows that *cad2* and *pad2* mutants display the same primary root growth as the wild type in our growth conditions, and that the primary root responds normally to both IAA and IBA. In the same way, BSO treatment (1 mM) affects neither primary root growth nor its response to IAA or IBA.

As expected, both IAA and IBA also induce LR density in the mature zone of the root in 10-day-old seedlings (Fig. 1B). LR density in both *cad2* and *pad2* mutants responds normally to IAA treatment but displays hyposensitivity to exogenous IBA. As expected, the addition of BSO phenocopies *cad2* and *pad2* mutants (Supplementary Fig. S2A). Finally, we could revert *cad2* hyposensitivity to IBA by adding exogenous GSH, while it has no effect on the sensitivity of wild-type plants to IBA (Supplementary Fig. S2B). Because the final emerged LR density depends both on LR initiation and subsequent LRP development, we also addressed the LRP density in the same conditions. Figure 1C reveals that both LRPs and emerged LR densities display IBA hyposensitivity in plants with low glutathione levels, suggesting that glutathione affects LR development upstream of LRP development. Another way to investigate LRP development is to force and synchronize LRP initiation by gravistimulation (Péret *et al.*, 2012). We therefore gravistimulated glutathione-deficient plants, in the absence or presence of exogenous IBA (10 µM). We observe that exogenous IBA treatment slightly slows down wild-type LRP development upon gravistimulation in our growth conditions (Fig. 1D). We also observe that *cad2*, *pad2*, and BSO-treated plants respond to IBA in a similar way to the wild type. This suggests that, once established, the regulation of LRP development by IBA is independent of glutathione, at least in its later stages. Taken together, these results tend to support a role for glutathione in IBA-dependent specification or activation of founder cells necessary for the initial establishment of LRPs.

Finally, we monitored RH elongation responses to IBA and IAA in glutathione-deficient plants (Fig. 1E, F). As for LRs, we notice that *cad2* and *pad2* mutants are hyposensitive to the IBA-dependent induction of RH growth but respond normally to IAA. Similarly, 1 mM BSO treatment also reduces the RH response to IBA but not IAA. We can therefore conclude that glutathione is also required for the IBA-dependent induction of RH growth.

### Glutathione levels affect auxin signalling in the basal part of the meristem

**Fig. 2.**
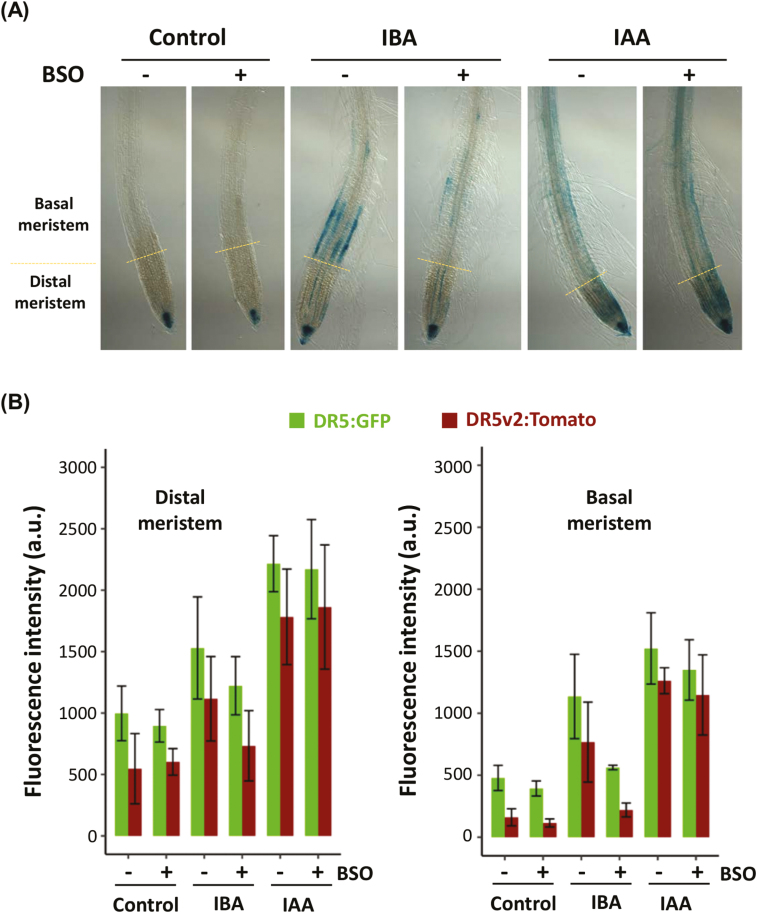
Glutathione is critical for the IBA-derived IAA signalling in the basal meristem. (A) Representative pictures of 8-day-old GUS-stained *DR5:GUS* plants grown on standard 1/2 MS medium (Control) or in the presence of 0.5 mM BSO, after a 24 h treatment with 10 µM IBA or 50 nM IAA (*n*≥7). (B) Quantification of GFP and Tomato fluorescent signals from 8-day-old plants expressing the *DR5:n3EGFP/DR5v2:ntdTomato* double reporter, and grown in the same conditions as (A) (*n*≥7).

We have shown that glutathione deficiency alters RH elongation and LRP density in response to IBA. We know that root tip-derived IAA transits through the LR cap to regulate both founder cell positioning and RH growth in the basal part of the meristem. We therefore investigated auxin response in the root tip, by using the *DR5:GUS* auxin signalling reporter (Ulmasov *et al.*, 1997). In standard growth conditions, we only reveal GUS staining in the quiescent centre and the columella, and BSO treatment by itself does not change the *DR5:GUS* expression pattern (Fig. 2A). As expected, exogenous IAA treatment for 24 h induces auxin response in the whole root tip and root epidermis, and the presence of BSO again has no effect on this distribution. In response to IBA, GUS staining increases in the distal meristem and strongly appears specifically in the trichoblast epidermal cell files in the basal part of the meristem, up to the differentiation zones. In the presence of BSO, this IBA-dependent strong signal in the basal meristem almost disappears while the signal in the distal meristem remains strong.

In order to confirm these results, we used another auxin signalling marker that allows the quantification of the auxin response, the *DR5:n3EGFP/DR5v2:ntdTomato* double reporter line (Liao *et al.*, 2015). We quantified both green fluorescent protein (GFP) and Tomato fluorescence in the distal and basal parts of the meristem (Fig. 2B). In agreement with the *DR5:GUS* reporter line, both IBA and IAA treatments increase auxin signalling in the distal and basal parts of the meristem. The presence of BSO does not significantly alter auxin signalling, either in standard conditions or upon exogenous IAA treatment. However, we can confirm that BSO severely represses IAA signalling in the basal part of the meristem in response to exogenous IBA, while it has limited effect in the distal part.

Hence we show that glutathione is specifically required for IBA-derived IAA signalling in the basal part of the meristem, where LR founder cells are specified and RH growth is determined. Moreover, we show that glutathione does not affect IAA signalling components since auxin signalling reporters respond normally to exogenous IAA when glutathione content is depleted.

### IBA-derived IAA responses are specifically affected by glutathione deficiency

**Fig. 3.**
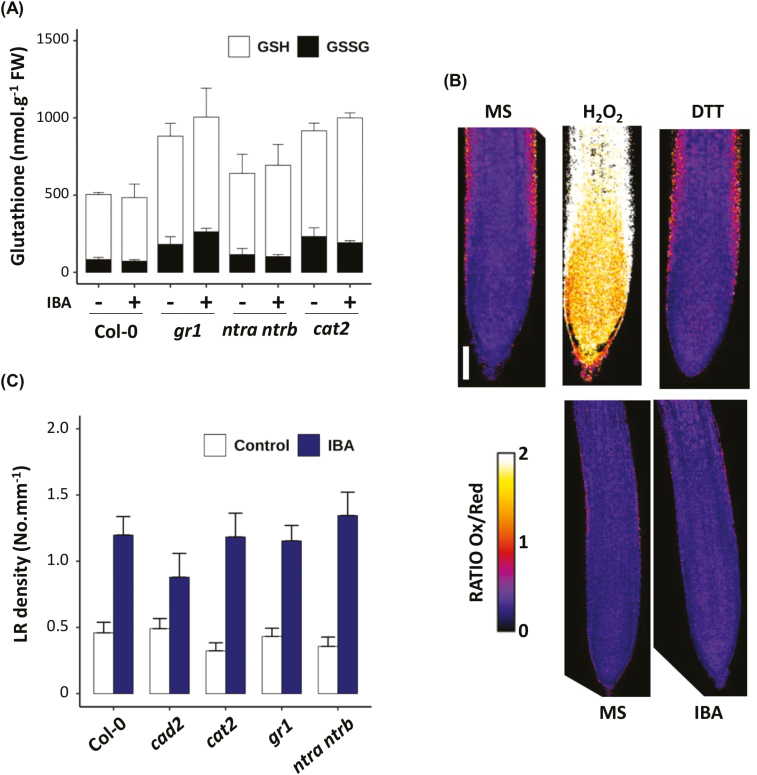
IBA hyposensitivity phenotype is specific to GSH-depleted plants. (A) Glutathione content in 8-day-old wild-type (Col-0), *cad2*, *gr1*, *ntra ntrb*, or *cat2* seedlings grown on standard 1/2 MS medium. Data represent the means of three biological repetitions. Reduced glutathione is represented in white (GSH) and oxidized glutathione in black (GSSG). (B) Representative pictures (*n*>10) of the roGFP2 reporter line grown on standard 1/2 MS medium, submitted to a 30 min treatment with 1 mM H_2_O_2_ then to an additional 30 min treatment with 10 mM DTT (top panel). The bottom panel represents roGFP2 reporter plants grown on standard 1/2 MS medium supplemented (IBA) or not (MS) with 10 µM IBA. Pictures are made from the ratio between the oxidized roGFP2 signal (excitation at 405 nm) and the reduced roGFP2 signal (excitation at 488 nm). Ratio values are represented in the color scale. (C) Emerged lateral root density of 10-day-old wild-type (Col-0), *cad2*, *cat2*, *gr1*, or *ntra ntrb* plants grown on standard 1/2 MS medium or in the presence of 10 µM IBA (*n*>16). Histograms represent the mean, and error bars represent the SD. Asterisks indicate a significant difference, based on a two-tailed Student *t*-test (*P<0.01; **P<0.001).

Because glutathione is a critical regulator of cellular redox homeostasis, we addressed root responses to IBA in other redox-related mutants. We chose to analyse the *gr1* mutant, affected in cytosolic and peroxisomal glutathione reduction (Marty *et al.*, 2009), the *cat2* mutant, affected in H_2_O_2_ detoxification (Queval *et al.*, 2007), and the *ntra ntrb* double mutant, affected in the thioredoxin-dependent thiol reduction system (Reichheld *et al.*, 2007). Quantification of both GSH and GSSG (Fig. 3A) shows that *gr1*, *ntra ntrb*, and *cat2* mutant plants have higher total glutathione concentrations than wild-type plants. The application of exogenous IBA has almost no effect on both GSH and GSSG levels. As expected, *cat2* and *gr1* mutants also display a higher glutathione oxidation status. In addition, the use of the roGFP2 probe allowed us to confirm that the presence of IBA does not generate any imbalance in the glutathione redox status in root tissues (Fig. 3B; Supplementary Fig. S3). In contrast to *cad2*, none of the other mutants display any LR density hyposensitivity to IBA (Fig. 3C). These results suggest that root responses to IBA specifically depend on glutathione overall levels rather than glutathione redox status or any general redox imbalance.

### IAA transport from the distal to the basal meristem is not targeted by glutathione

We have presented data showing that the IBA-derived IAA response in the basal meristem is dependent on glutathione levels. AUX1 and PIN2 are the IAA transporters that ensure the apico-basal IAA flux in the root cap and the root tip epidermis. We previously published our results showing that strong BSO treatment induces a long-term decrease in the expression of *AUX1* and *PIN2* (Bashandy *et al.*, 2010).

**Fig. 4.**
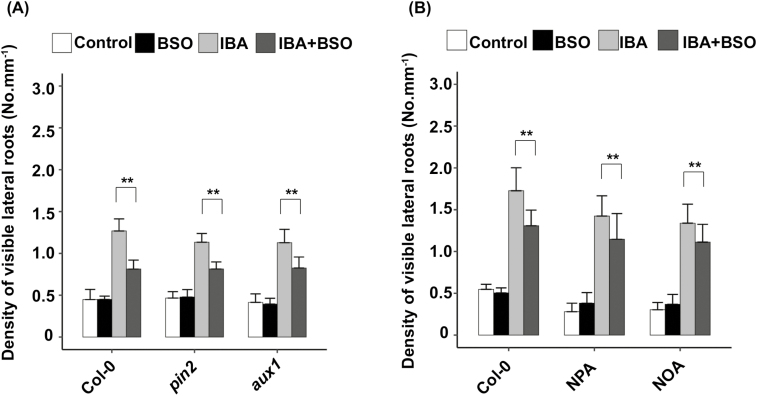
IAA transport is not the target of glutathione. (A) Emerged lateral root density of 10-day-old wild-type (Col-0), *aux1*, or *pin2* plants grown on standard 1/2 MS medium, or in the presence of different combinations of 0.5 mM BSO and 10 µM IBA (*n*>10). (B) Emerged lateral root density of 10-day-old wild-type plants(Col-0) grown on standard 1/2 MS medium, supplemented or not with the transport inhibitors NPA (1 µM) or NOA (1 µM) (*n*≥15). Histograms represent the mean, and error bars represent the SD. Asterisks indicate a significant difference, based on a two-tailed Student *t*-test (**P<0.001).

In order to determine if *AUX1* and/or *PIN2* are the targets of glutathione to modulate root responses to IBA, we examined LR density in *aux1* and *pin2* mutants. As shown in Fig. 4A, *aux1* and *pin2* mutants still display hyposensitivity to IBA upon BSO treatment. In other words, the glutathione regulation still occurs in both *aux1* and *pin2* mutants, revealing that neither AUX1 nor PIN2 is regulated by glutathione to control root responses to IBA. Because AUX1 and PIN2 are members of multigene families, we wanted to ensure that other members of AUX/LAX or PIN families are not replacing AUX1 and PIN2 in the respective mutants. We therefore addressed IBA responses with or without BSO treatment in the presence of specific inhibitors of both families (Fig. 4B). We observed that BSO still leads to LR hyposentitivity to IBA both in the presence of *N*-1-naphthylphthalamic acid (NPA), that inhibits PIN-dependent auxin efflux, and in the presence of 1-naphthoxyacetic acid (NOA), a specific inhibitor of AUX/LAX influx facilitators. All together, these data suggest that the glutathione-dependent control of root responses to IBA does not affect IAA transport from the root apex to the basal meristem.

### Looking for glutathione targets in IBA pathways.

**Fig. 5.**
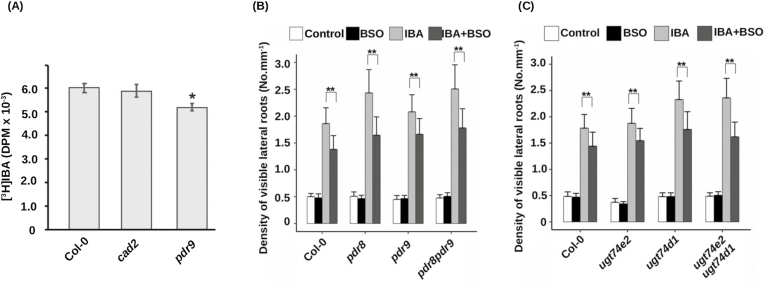
IBA homeostasis does not display sensitivity to glutathione. (A) Uptake of [^3^H]IBA by wild-type (Col), *cad2-1*, or *pdr9-1* excised root tips of 8-day-old plants. Data represent eight replicates with five root tips per genotype and per replicate. (B) Emerged lateral root density of 10-day-old wild-type (Col-0), *pdr8*, *pdr9*, or *pdr8 pdr9* plants grown on standard 1/2 MS medium, or in the presence of different combinations of 0.5 mM BSO and 10 µM IBA (*n*≥15). (C) Emerged lateral root density of 10-day-old wild-type (Col-0), *ugt74e2*, *ugt74d1*, or *ugt74e2 ugt74d1* plants grown on standard 1/2 MS medium, or in the presence of different combinations of 0.5 mM BSO and 10 µM IBA (*n*≥15). Histograms represent the mean, and error bars represent the SD. Asterisks indicate a significant difference, based on a two-tailed Student *t*-test (*P<0.05; **P<0.001).

Since IAA transport was not the target of glutathione, we investigated IBA homeostasis in plants with low glutathione content. Interestingly, *gsh1* mutants display pleiotropic phenotypes opposite to the phenotypes of mutant alleles in *PDR8/PEN3/ABCG36* for IBA sensitivity, but also for sensitivity to *Pseudomonas* and cadmium treatments (Kim *et al.*, 2007; Strader and Bartel, 2009; Lu *et al.*, 2015). ABCG36, together with its homologue PDR9/ABCG37, is responsible for IBA efflux from the cells, which prompted us to investigate the IBA import into plant cells in the *cad2* mutant. As shown in Fig. 5A, we did not detect any impairment in [^3^H]IBA accumulation in *cad2* excised root tips, in contrast to the expected reduction of uptake in a gain-of-function allele of *PDR9* (*pdr9-1*). This suggests that IBA uptake from the medium is not perturbed by glutathione deficiency. This is consistent with the normal response of primary root growth to IBA in such conditions (Supplementary Fig. S1A, B).

In order to better investigate IBA transport, we analysed the glutathione-dependent LR and RH response to IBA in *abcg36/pdr8* and *abcg37/pdr9* mutants (Fig. 5B; Supplementary Fig. S4). As expected, the *pdr8* mutant displays hypersensitivity to exogenous IBA. However, both mutants are still clearly resistant to IBA in the presence of BSO. Because of putative redundancy between these two proteins, we generated the *pdr8 pdr9* double mutant that displays a *pdr8*-like hypersensitivity of LR density to exogenous IBA. As for single mutants, LR density and RH length in the double mutant are induced by IBA, but BSO is still able to decrease this response. This result means that IBA efflux transporters ABCG36 and ABCG37 are not the targets of glutathione-dependent regulation.

We know that IBA homeostasis is also regulated via conjugation with glucose, and several glycosyltransferases are able to catalyse such a reaction (Jackson *et al.*, 2001; Tognetti *et al.*, 2010; Jin *et al.*, 2013; Zhang *et al.*, 2016). Among them, we assayed *ugt74d1* and *ugt74e2* mutants. Again, we could observe that LR and RH responses to IBA are still BSO sensitive in both mutants, although *ugt74d1* LR density is hypersensitive to IBA compared with the wild type (Fig. 5C; Supplementary Fig. S4). Because of putative redundancy between these two glycosyltransferases, we generated a *ugt74e2 ugt74d1* double mutant. However, the response to IBA is still reduced in the presence of BSO in the double mutant, suggesting that these glycosyltransferases are not the targets of glutathione-dependent regulation.

**Fig. 6.**
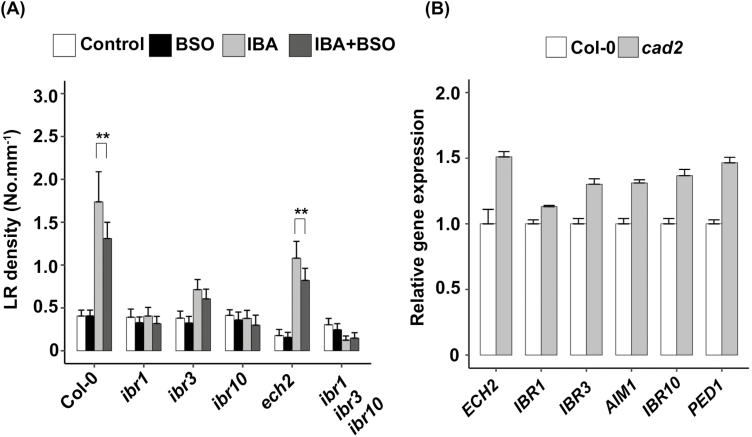
IBA conversion to IAA does not display sensitivity to glutathione. (A) Emerged lateral root density of 10-day-old wild-type (Col-0), *ibr1*, *ibr3*, *ibr10*, *ibr1 ibr3 ibr10*, or *ech2* plants grown on standard 1/2 MS medium, or in the presence of different combinations of 0.5 mM BSO and 10 µM IBA (*n*≥15). (B) Expression levels (qRT-PCR) of *IBR1*, *IBR3*, *IBR10*, *ECH2*, *AIM1*, and *PED1* genes in *cad2* relative to wild-type 8-day-old plants grown on standard 1/2 MS medium. Data represent three independent replicates with 20 plants per replicate. Histograms represent the mean, and error bars represent the SD. Asterisks indicate a significant difference, based on a two-tailed Student *t*-test (*P<0.01; **P<0.001).

Finally, we also investigated the enzymatic pathway involved in the IBA to IAA conversion in the peroxisome. Figure 6A shows that *ibr1*, *ibr10*, and *ibr1 ibr3 ibr10* mutants are fully insensitive to exogenous IBA in our growth conditions, thus making it impossible to genetically address the putative dependency of IBR1 and IBR10 on glutathione levels. However, we noticed that in *ech2* and *ibr3* mutants, LRs are still hyposensitive to IBA in the presence of BSO. Again, this suggests that ECH2 and IBR3 are not regulated in a glutathione-dependent manner. In order to detect an eventual defect in gene expression in glutathione-deficient plants, we analysed the expression level of genes involved in IBA to IAA conversion (Fig. 6B). Surprisingly, *ECH2*, *IBR1*, *IBR3*, *IBR10*, *AIM1*, and *PED1* genes were all moderately (10–50% increase) but consistently up-regulated in the *cad2* mutant compared with the wild type. In any case, this does not allow us to identify any target that could be transcriptionally down-regulated upon glutathione depletion.

To conclude, we carefully examined most of the known components of IBA homeostasis and response pathways, but none of them seems to be the target of glutathione-dependent regulation.

### IBA and glutathione control RH responses to phosphate deprivation

**Fig. 7.**
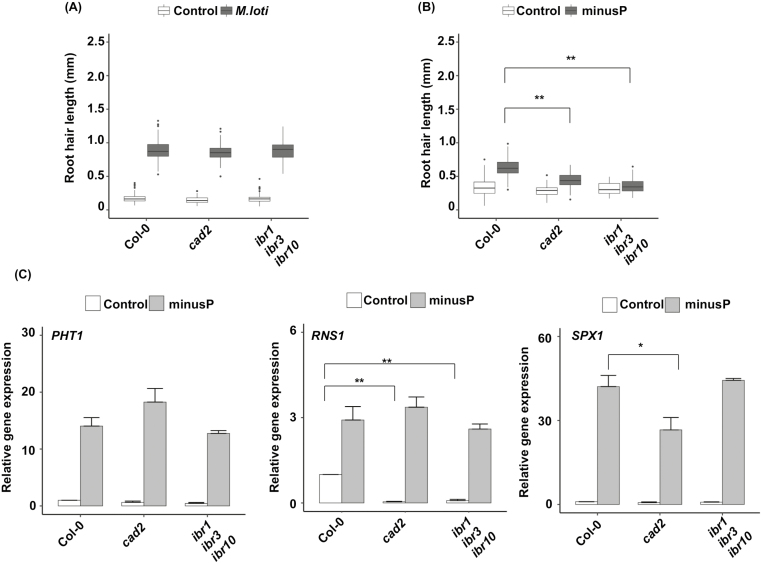
Glutathione and IBA are specifically required for RH growth response to low phosphate. (A) RH length in 8-day-old wild-type (Col-0), *cad2*, or *ibr1 ibr3 ibr10* plants grown on standard 1/2 MS medium in the absence or the presence of the PGPR *Mesorhizobium loti* (see the Materials and methods). (B) RH length in 8-day-old wild-type (Col-0), *cad2*, or *ibr1 ibr3 ibr10* plants grown on 3/10 MS medium supplemented with 500 µM sodium phosphate (control) or 500 µM sodium chloride (minusP). (C) Expression levels (qRT-PCR) of *PHT1;4*, *RNS1*, and *SPX1* low-phosphate-responsive genes, relative to the *ACT2* control gene, in 8-day-old wild-type (Col-0), *cad2*, or *ibr1 ibr3 ibr10* plants grown on 1/3 MS medium supplemented with 500 µM NaH_2_PO_4_ (control) or 500 µM NaCl (minusP). Data represent three independent replicates with 20 plants per replicate and are normalized relative to the wild-type value in control conditions. Histograms represent the mean, and error bars represent the SDC. Asterisks indicate a significant difference, based on a two-tailed Student *t*-test (*P<0.01; **P<0.001).

We analysed the RH elongation rate in response to phosphate starvation and to *M. loti*. We observed that both stimuli increase RH length in wild-type plants (Fig. 7A, B). Both *cad2* and *ibr1 ibr3 ibr10* mutants display a wild-type-like RH response to *M. loti*, suggesting that neither glutathione nor IBA participates in RH elongation response to PGPR (Fig. 7A).

In contrast, we observed that the *cad2* mutant is clearly hyposensitive to phosphate deprivation, and that the RH elongation response to phosphate starvation is almost fully abolished in the *ibr1 ibr3 ibr10* mutant (Fig. 7B). We also confirmed the decreased glutathione levels in the *cad2* mutant while the *ibr1 ibr3 ibr10* mutant does not have an altered glutathione content and redox status (Supplementary Fig. S5). Interestingly, phosphate deprivation does not significantly affect total glutathione content in plants but increases the GSH/GSSG ratio.

We examined if *cad2* and *ibr1 ibr3 ibr10* mutants affect the general response to phosphate deficiency or specifically the response of RHs to this abiotic stress. We therefore quantified the expression of marker genes known to be induced in response to phosphate deprivation. Figure 7C shows that the induction of the expression of marker genes in response to phosphate starvation is not abolished in *cad2* and *ibr1 ibr3 ibr10* mutants. We only noticed a slight down-regulation of *SPX1* in the *cad2* mutant. As *SPX1* encodes a negative regulator of phosphate starvation responses, this could explain why other responsive genes are slightly up-regulated in *cad2*.

We can conclude that both IBA conversion to IAA and glutathione are required to trigger the root hair response to phosphate starvation, without affecting general responses to this abiotic stress.

## Discussion

### Glutathione regulation of IBA homeostasis or conversion to IAA in the root cap

In this work, we first report that RH and LR responses to exogenous IBA, but not IAA, are impaired by glutathione deficiency. More precisely, the glutathione-dependent control of IBA response affects early steps of LR development, either specification of founder cells or their activation to divide and form an LRP. We also show that low glutathione levels alter IBA-dependent auxin signalling in the basal meristem, where RH elongation occurs and LR founder cells are specified. Finally, we show herein that transporters known to be involved in the apico-basal flux of IAA to the basal meristem, namely AUX1 and PIN2, are not the targets of the glutathione-dependent control. All these data strongly suggest that glutathione modulates IBA homeostasis or IBA to IAA conversion in the root cap, although it does not exclude that the same regulation can also occur in additional tissues.

All the phenotypes reported here concern root responses to exogenous IBA, and one can wonder if the glutathione-dependent regulation normally occurs in physiological conditions, modulating pathways relying on endogenous IBA. Because of our scarce knowledge of IBA metabolism and the absence of genetic tools concerning IBA biosynthesis, it is rather difficult to address such a question. However, we accumulated several clues that, taken together, strongly support the importance of glutathione for the control of endogenous IBA pathways. First, we observe in *cad2* mutant seedlings grown under standard conditions (i.e. without exogenous IBA) a general increase in the expression of all genes involved in IBA to IAA conversion (Fig. 6B). This might reveal a feedback mechanism that would report an excess of IBA or a depletion in IBA-derived IAA in low glutathione conditions. Secondly, we observe that both *ugt* and *pdr* double mutants are hypersensitive to exogenous IBA (Fig. 5B, C), which is consistent with the already reported overaccumulation of endogenous free IBA in corresponding single mutants (Strader and Bartel, 2009; Tognetti *et al.*, 2010; Ruzicka *et al.*, 2010). Interestingly, it appears that BSO has more effect in both *ugt* and *pdr* mutant genotypes than in wild-type plants, reducing IBA response by 20–25% in wild-type plants, but by 28–33% in *pdr* and *ugt* double mutants (Fig. 5B, C). This supports a role for glutathione in modulating responses to endogenous IBA. Finally, we show that both *cad2* and the *ibr1 ibr3 ibr10* triple mutant display hyposensitivity in their RH response to phosphate, but not to *M. loti*. In addition, neither is impaired in the general plant response to phosphate deprivation, but only in the RH elongation response. This set of arguments proves that both the endogenous IBA pathway and glutathione mediate RH response to phosphate starvation, and strongly suggests that the glutathione-dependent regulation of the endogenous IBA pathway is modulating the RH response.

### Auxins fine-tune of root architecture in response to phosphate availability

Although well known to play important roles in plant development, the function of IBA as a source of IAA relative to other IAA sources, such as *de novo* synthesis or conjugated forms, is still very mysterious and we might wonder ‘Why do plants need more than one type of auxin?’ (Simon and Petrášek, 2011; Frick and Strader, 2018). A previous work has reported that IBA regulates both plant development and resistance to water stress, suggesting an important function for IBA in adapting plant development to abiotic stress (Tognetti *et al.*, 2010). However, to our knowledge, our work is the first to clearly demonstrate that an IBA-dependent pathway is necessary for IAA to adapt some developmental pathway, namely RH elongation, to a specific abiotic stress (phosphate starvation).

In their recent work, Bhosale *et al.* (2018) already revealed the increase in IAA levels in the root cap in response to low external phosphate, necessary for RH elongation. However, because they observed a significant increase in *TAA1* (*TRYPTOPHAN AMINOTRANSFERASE OF ARABIDOPSIS 1*) gene expression in such conditions, they hypothesized that the increase in IAA levels in the root cap is due to the induction of an indole-3-pyruvic acid (IPyA)-dependent pathway, the main one responsible for *de novo* IAA synthesis in Arabidopsis (Mashiguchi *et al.*, 2011). Intriguingly, RH elongation response to external low phosphate is almost completely abolished in both *ibr1 ibr3 ibr10* and *taa1* mutants (Fig. 7B; fig. 2B and C in Bhosale *et al.*, 2018). One could imagine that two independent routes of IAA synthesis, namely the *TAA1*-dependent *de novo* synthesis and the IBA to IAA conversion, are both critical and induced in the root cap in response to phosphate deprivation. IBA can itself be derived from IAA and we can imagine that an increase in IBA to IAA conversion would require an increase in IBA levels, and therefore more IAA to supply the IBA stock. However, thinking that a root cap increase in IAA to increase IBA in order to increase IAA makes almost no sense. Thus, we suggest that external low phosphate induces *TAA1* expression in the whole plant to increase global available auxin levels on the one hand, and on the other hand activates the local IBA to IAA conversion in the root cap to ensure a local appropriate developmental response.

However, we must remember that we are working *in vitro* with homogenous media containing a given concentration of phosphate. A very elegant recent work has reported that, when subjected to a heterogenous environment, using a Dual-flow-RootChip, a single root increases RH length in the medium having the highest phosphate concentration (Stanley *et al.*, 2018). This observation contrasts with the induction of RH growth by low phosphate in homogenous *in vitro* media. RH adaptation to phosphate probably results from the crosstalk between systemic information based on overall available phosphorus in the plant, and local information based on phosphate availability in the environment, that we cannot differentiate in our *in vitro* homogenous conditions. Such dual control has been extensively documented for root responses to another important nutrient, nitrogen (Ruffel and Gojon, 2017). We might therefore envisage that both sources of IAA are differentially regulated by systemic and local signals. This might ensure a very acute dosage of free IAA to optimize root adaptation to the plant’s metabolism and environment. In such a context, glutathione could be a central regulator of local IBA to IAA adaptation to external phosphate in the root cap.

### Unravelling the glutathione-dependent regulation of the IBA pathway

Although we explored most of the known components of IBA pathways (Supplementary Fig. S6), we were not able to identify how glutathione regulates IBA homeostasis or conversion to IAA. This can first be explained by the difficulties of working with IBA which is present in low amounts in plants compared with IAA, and is therefore difficult to detect and quantify (Frick and Strader, 2018). Moreover, many components of the IBA pathways remain to be identified. Undoubtedly, the development of new tools, such as IBA-specific reporters or probes, or mutants in IBA biosynthesis, would be very helpful to decipher the important roles played by this auxin in plants. The other problem we faced is the complex multifaceted roles of glutathione in cells, that prevented us from trying to decipher the mechanism from the glutathione starting point. The only clue we get is that IBA hyposensitivity is specific to plants with reduced amounts of glutathione but does not occur in plants affected in other redox pathways such as glutathione or thioredoxin reduction (Fig. 3). This suggests that IBA regulation by glutathione may act through glutathionylation or via the activity of thiol reductases that specifically depend on glutathione, such as glutaredoxins. One hypothesis would be that IBA, or a precursor, could itself be glutathionylated, which would be responsible for its storage or transport. In Arabidopsis, the Tau class of glutathione *S*-transferase (GSTU) has been shown to glutathionylate some fatty acids, ranging from short to long acyl chains (Dixon and Edwards, 2009). Similarly, GSTU19 and GSTF8 have been proposed to glutathionylate 12-oxo-phytodienoic acid (OPDA), hence allowing its translocation from the chloroplast to the peroxisome where it is converted to jasmonic acid (Davoine, 2006; Dixon and Edwards, 2009). Interestingly, among the 28 members of the Tau class, encoding cytosolic enzymes, several *GSTU* genes are transcriptionally regulated by IBA in the Arabidopsis root tip (Xuan *et al.*, 2015) and/or by phosphate deficiency in Arabidopsis roots (Lin *et al.*, 2011), and could be good candidates. It would be interesting to assay the IBA responses of RHs and LRs in corresponding mutants.

Because IBA to IAA conversion occurs in the peroxisome, we might also envisage that the glutathione-dependent regulation is not specific to IBA pathways but affects the peroxisomal machinery. One of the main peroxisomal functions is the β -oxidation of fatty acids, which is essential to supply energy during seed germination. We never observed any defect in seed germination in *cad2* or *pad2* mutants, in contrast to mutants in peroxisomal functions which generally strongly affect seed germination. However, we cannot exclude that the glutathione-dependent regulation only occurs in the root cap and therefore mainly affects IBA to IAA conversion although regulating the peroxisome machinery. Among the PEROXIN proteins, many participate in the peroxisomal matrix protein import machinery (Cross *et al.*, 2016). PEX5 is a central cargo protein that recognizes proteins targeted with the specific PTS1 (Peroxisomal Targeting Signal 1) signal peptide and import them into the peroxisome. PEX7 recognizes proteins harbouring another signal peptide (PTS2), and then binds to PEX5 for import into the peroxisome. Interestingly, previous work on human and *Pichia pastoris* PEX5 revealed that its activity and oligomerization depend on a redox switch affecting a conserved N-terminal cysteine (Ma *et al.*, 2013; Apanasets *et al.*, 2014). However, such regulation is not specific to glutathione but rather depends on the redox status and the content of ROS of peroxisomes.

Finally, the last hypothesis would be that glutathione regulates IBA homeostasis or conversion to IAA via an as yet unidentified or not assayed component. Quantification of IBA to IAA conversion would be helpful in order to confirm this hypothesis. Concerning the IBA to IAA β -oxidation pathway in the peroxisome, PED1 has been reported to harbour a redox-sensitive switch affecting a conserved cysteine and that participates in activating the enzyme when reduced (Pye *et al.*, 2010). In contrast to most of the other enzymes involved in the conversion pathway, PED1 is targeted to the peroxisome through a PTS2 signal peptide. It is interesting to notice that *pex5-1*, in contrast to *pex5-10*, is not altered in PTS1-dependent import but only in PTS2-dependent import of proteins into the peroxisome (Woodward and Bartel, 2005; Khan and Zolman, 2010). *pex5-1*, although not displaying any phenotype during germination, is highly affected in IBA responses. This might reveal that IBA to IAA conversion is highly dependent on a PTS2-targeted protein, and PED1 could therefore be a good candidate to assay. However, PED1 is not specific to the IBA to IAA pathway since it acts in almost all β -oxidation pathways occurring in the peroxisome. We could imagine a local glutathione-dependent regulation of PED1 activity specifically in the root cap.

In conclusion, our work points out the important role of glutathione in both controlling IBA to IAA conversion required for root development and in mediating developmental responses to nutrient deprivation. This opens up novel perspectives in understanding how redox homeostasis can signal and integrate environmental constraints to trigger developmental adaptations via the regulation of morphogenetic hormonal pathways.

## Supplementary data

Supplementary data are available at *JXB* online.

Fig. S1. Glutathione does not affect primary root response to IBA.

Fig. S2. Glutathione regulates LR responses to IBA.

Fig. S3. IBA does not alter redox status of GSH in root tips.

Fig. S4. RH response to IBA in mutants affected in IBA homeostasis.

Fig. S5. Glutathione content in response to low phosphate.

Fig. S6. Auxin-related tools used in this study.

Table S1. List of primers used for qPCR analyses.

eraa195_suppl_Supplementary_Figures_S1-S6_Table_S1

## Abbreviations

BSO: buthionine sulfoximine
GSH: reduced glutathione
GSSG: oxidized glutathione
IAA: indole acetic acid
IBA: indole butyric acid
LR: lateral root
LRP: lateral root primordium
MS: Murashige and Skoog
NOA: 1-naphthoxyacetic acid
NPA: *N*-1-naphthylphthalamic acid
PGPR: plant growth-promoting rhizobacteria
RH: root hair
ROS: reactive oxygen species
