# Supplementary material for: A glutathione-dependent control of the indole butyric acid pathway supports Arabidopsis root system adaptation to phosphate deprivation
Source: J Exp Bot. 2020 Apr 20;71(16):4843–57. doi: 10.1093/jxb/eraa195 (PMC7410191; doi:10.1093/jxb/eraa195)
Supplement: eraa195_suppl_Supplementary_Figures_S1-S6_Table_S1 [file eraa195_suppl_supplementary_figures_s1-s6_table_s1.pdf]

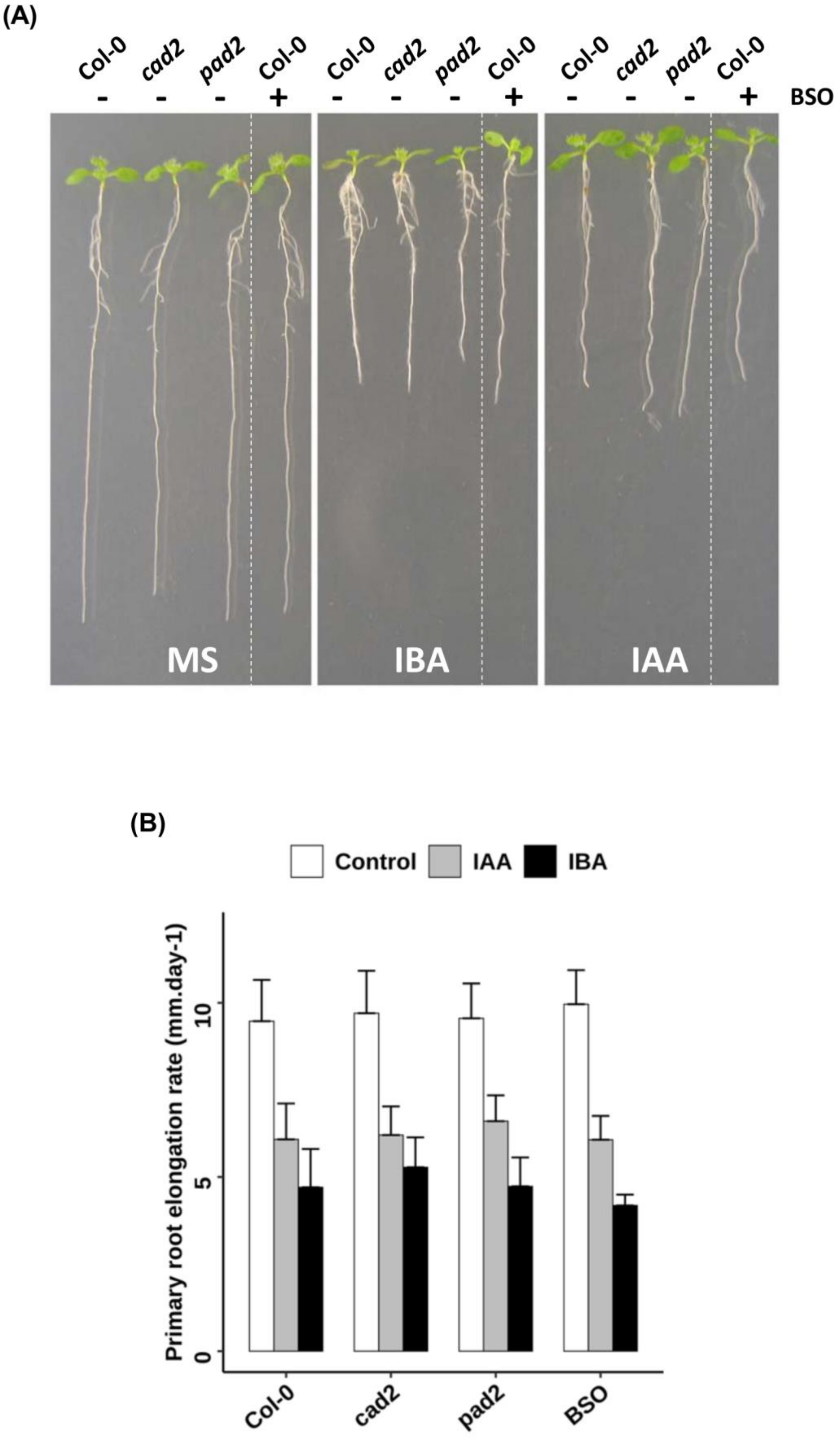
(A)
Col-0
cad2
pad2
Col-0
Col-0
cad2
pad2
Col-0
Col-0
cad2
pad2
Col-0
- - - + - - - + - - - + BSO
MS
IBA
IAA
(B)
Control
IAA
IBA
Primary root elongation rate (mm.day-1)
0
5
10
Col-0
cad2
pad2
BSO

Supplementary Figure 1. Glutathione does not affect primary root response to IBA.

A. Pictures of representative 8-day-old wild-type, *cad2*, and *pad2* plants grown on standard ½ MS medium supplemented or not with 10 µM IBA or 50 nM IAA. Wild-type plants grown on 0.5 mM BSO are also presented.
B. Quantification of primary root elongation rate of 8-day-old wild-type (Col-0), *cad2* or *pad2* mutant plants grown on standard ½ MS medium (Control), or in the presence of 50 nM IAA (IAA) or 10 µM IBA (IBA). Wild-type plants in the presence of 0.5 mM BSO (BSO) were also assayed (n≥12).

Histograms represent the mean and error bars represent the standard deviation. Asterisks indicate a significant difference, based on a two-tailed Student t-test (*P<0,01; **P<0,001).

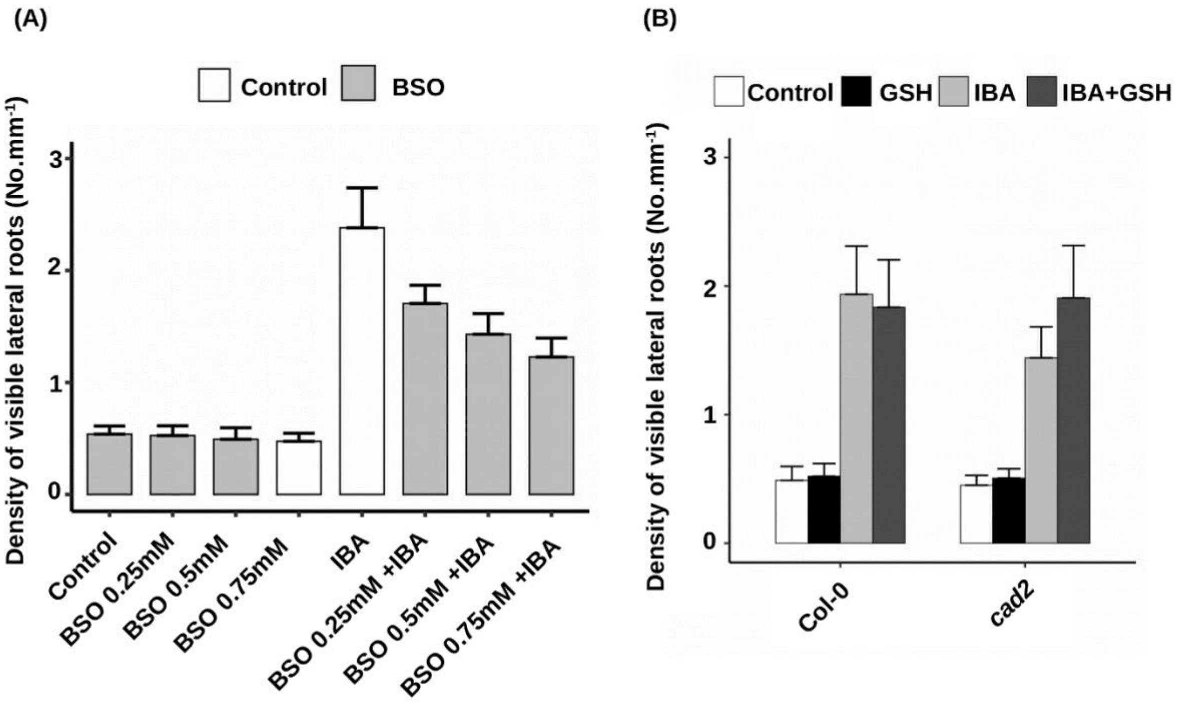

Supplementary Figure 2. Glutathione regulates LR responses to IBA.

A. Emerged LR density of 10-day-old wild-type plants grown on standard ½ MS medium (control), or in the presence of different concentrations of BSO, in the absence or the presence of IBA 10 µM (n≥15).
B. Emerged LR density of 10-day-old wild-type and *cad2* plants grown on standard ½ MS medium (control), or in the presence of IBA 10 µM and 5 mM GSH (n≥15).

Histograms represent the mean and error bars represent the standard deviation. Asterisks indicate a significant difference, based on a two-tailed Student t-test (*P<0,01; **P<0,001).

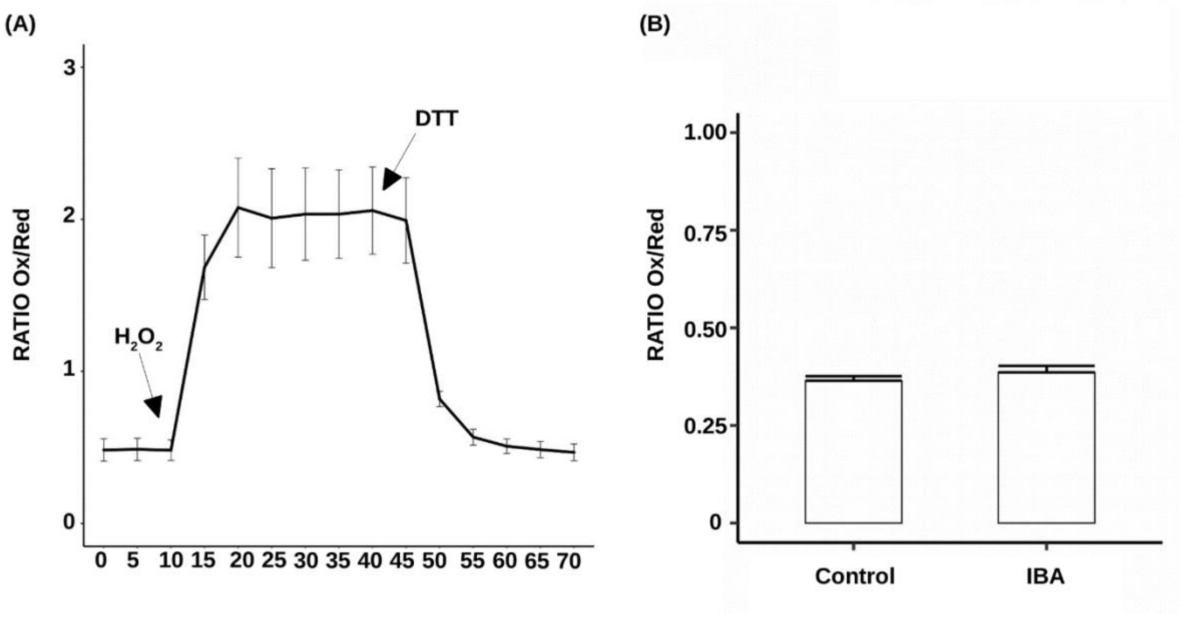

Supplementary Figure 3. IBA does not alter redox status of GSH in root tips.

A. Kinetics of roGFP2 reporter response to 1 mM H_2O_2 then to an additional 10 min treatment with 10 mM DTT. The ratio between the oxidised roGFP2 signal (excitation at 405 nm) and the reduced roGFP2 signal (excitation at 488 nm) is used to monitor the glutathione redox status.
B. Quantification of roGFP2 ratio in root tips from wild-type plants grown on standard ½ MS medium or in the presence of 10 µM IBA for 8 days.

Data represent the mean and error bars represent the standard deviation (n≥9).

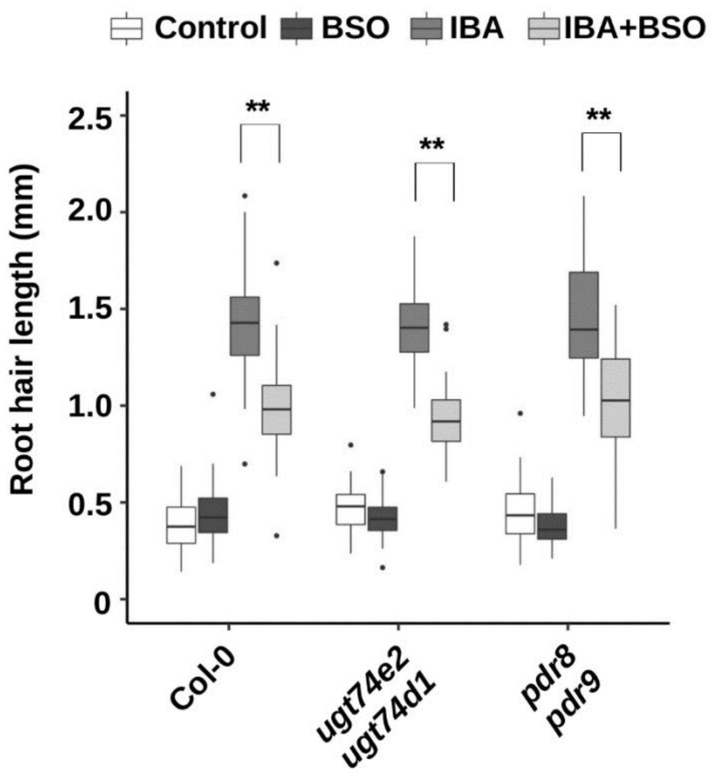

Supplementary Figure 4. RH response to IBA in mutants affected in IBA homeostasis.

RH length of 8-day-old wild-type (Col-0), *ugt74e2 ugt75d1* and *pdr8 pdr9* plants grown on standard ½ MS medium, or in the presence of different combinations of BSO 0.5 mM and IBA 10 µM (n≥50). Asterisks indicate a significant difference, based on a two-tailed Student t-test (**P<0,001).

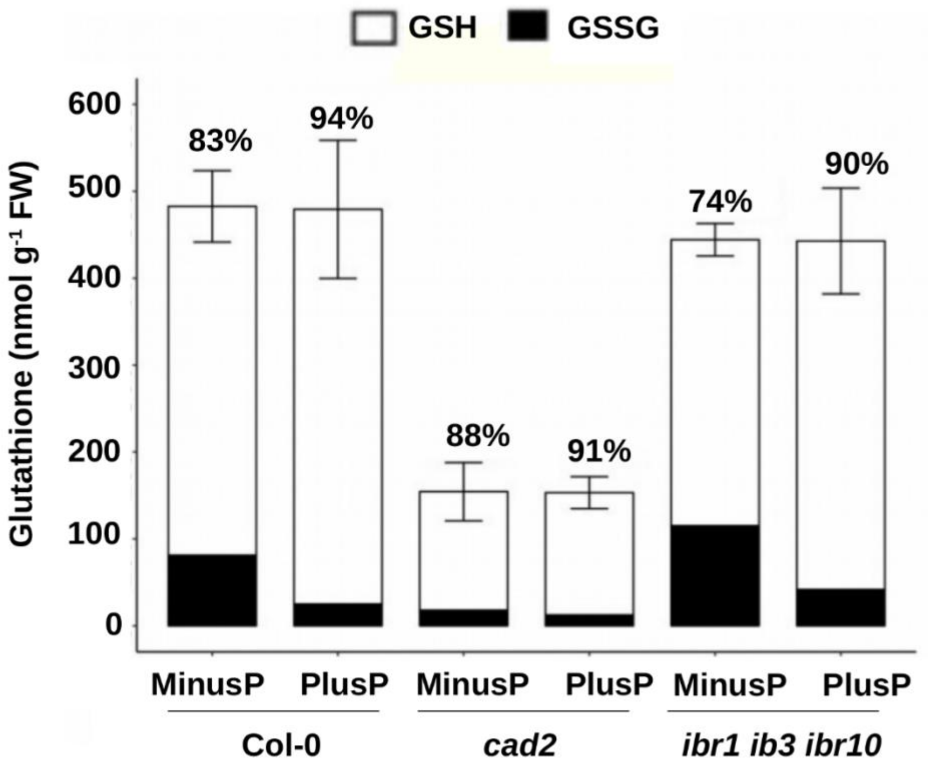

Supplementary Figure 5. Glutathione content in response to low phosphate.

Glutathione content in 8-day-old wild-type (Col-0), *cad2*, or *ibr1 ibr3 ibr10* seedlings grown on 1/3 MS medium supplemented with 500 µM NaH_2PO_4 (control) or 500 µM NaCl (minusP). Data represent the means of 3 biological repetitions. Reduced glutathione is represented in white (GSH) and oxidised glutathione in black (GSSG). Histograms represent the mean and error bars represent the standard deviation.

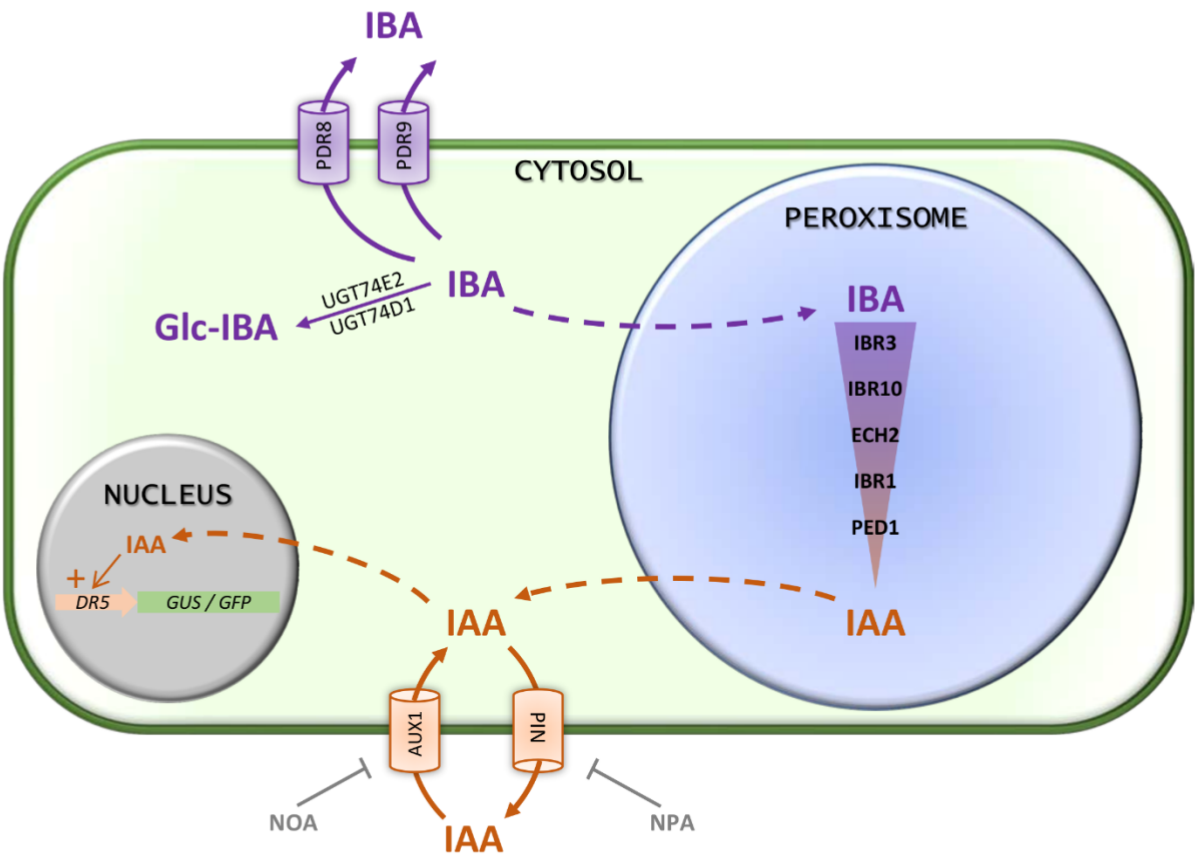

Supplementary Figure 6. Auxin-related tools used in this study.

This scheme represents the different genetic and biochemical tools used in our study. They concern IBA homeostasis, conjugation to IAA, IAA transport and signalling reporters. IAA: Indole Acetic Acid; IBA: Indole Butyric Acid; Glc-IBA: Glucosylated IBA; NOA: 1-NaphthOxyacetic Acid; NPA: N-1-NaphthylPhthalamic Acid.

Gene	5’ Primer	3’ Primer
ACTIN2	5’-CTTGCACCAAGCAGCATGAA-3’	5’-CCGATCCAGACACTGTACTTCCTT-3’
ACTIN7	5’-CCAGGAATTGCTGACCGTAT-3’	5’-GATTGATCCTCCGATCCAGA-3’
GAPDH	5’-TTGGTGACAACAGGTCAAGCA-3’	5’-AAACTTGTCGCTCAATGCAATC-3’
IBR1	5’-ATCACCGGTAGCTCTGAAGTGAGG-3’	5’-ATGTCTCCCGTTGTTCCCAACC-3’
IBR3	5’-TTCTCGCAATGGCCAAGGTTGC-3’	5’-GCTGCTCCATGAACTTGTATTGCC-3’
IBR10	5’-GCTTTCGATGCCGGATTATT-3’	5’-TTCCCACTCAACAACAGCTC-3’
ECH2	5’-ATGGGCAGGATTTGATCCAGGTC-3’	5’-ACTGCTGCCCATGCAACAAGAG-3’
AIM1	5’-GAAAGGCCCTCTCTTTGTTC-3’	5’-AATCACTGCCTCTATGACCA-3’
PED1	5’-AGGAGCGGTTCTCCTAATGA-3’	5’-CCTGAATACACCAAGAACGG-3’
PHT1;4	5’-CCTCGGTCGTATTTATTACCACG-3’	5’-CCATCACAGCTTTTGGCTCATG-3’
RNS1	5’-TGATGCCTCTAAACCATTCGAT-3’	5’-TACCATGCTTCTCCCATTCG-3’
SPX1	5’-CGGGTTTTGAAGGAGATCAG-3’	5’-GCGGCAATGAAAACACACTA-3’

Supplementary Table 1. List of primers used for qPCR analyses.
